# Prognostic nomogram to predict cancer-specific survival with small-cell carcinoma of the prostate: a multi-institutional study

**DOI:** 10.3389/fonc.2024.1349888

**Published:** 2024-05-10

**Authors:** Yupeng Di, Jiazhao Song, Zhuo Song, Yingjie Wang, Lingling Meng

**Affiliations:** ^1^ Department of Radiotherapy, Air Force Medical Center, PLA, Beijing, China; ^2^ Department of Radiation Oncology, Senior Department of Oncology, The Fifth Medical Center of PLA General Hospital, Beijing, China

**Keywords:** small-cell carcinoma, prostate, nomogram, cancer-specific survival, surveillance epidemiology and end results

## Abstract

**Objective:**

The aim of this study is to examine the predictive factors for cancer-specific survival (CSS) in patients diagnosed with Small-Cell Carcinoma of the Prostate (SCCP) and to construct a prognostic model.

**Methods:**

Cases were selected using the Surveillance, Epidemiology, and End Results (SEER) database. The Kaplan-Meier method was utilized to calculate survival rates, while Lasso and Cox regression were employed to analyze prognostic factors. An independent prognostic factor-based nomogram was created to forecast CSS at 12 and 24 months. The model’s predictive efficacy was assessed using the consistency index (C-index), calibration curve, and decision curve analysis (DCA) in separate tests.

**Results:**

Following the analysis of Cox and Lasso regression, age, race, Summary stage, and chemotherapy were determined to be significant risk factors (P < 0.05). In the group of participants who received training, the rate of 12-month CSS was 44.6%, the rate of 24-month CSS was 25.5%, and the median time for CSS was 10.5 months. The C-index for the training cohort was 0.7688 ± 0.024. As for the validation cohort, it was 0.661 ± 0.041. According to the nomogram, CSS was accurately predicted and demonstrated consistent and satisfactory predictive performance at both 12 months (87.3% compared to 71.2%) and 24 months (80.4% compared to 71.7%). As shown in the external validation calibration plot, the AUC for 12- and 24-month is 64.6% vs. 56.9% and 87.0% vs. 70.7%, respectively. Based on the calibration plot of the CSS nomogram at both the 12-month and 24-month marks, it can be observed that both the actual values and the nomogram predictions indicate a predominantly stable CSS. When compared to the AJCC staging system, DCA demonstrated a higher level of accuracy in predicting CSS through the use of a nomogram.

**Conclusion:**

Clinical prognostic factors can be utilized with nomograms to forecast CSS in Small-Cell Carcinoma of the Prostate (SCCP).

## Introduction

Prostate cancer has a subtype called small-cell carcinoma (SCCP), which is rare but extremely aggressive and malignant. It accounts for about 0.36% of newly detected cases (1, 2). The majority of patients (70% to 80%) are diagnosed with advanced disease. Chemotherapy is the mainstay of clinical treatment (3, 4). Nevertheless, a number of studies have demonstrated that radiation or surgical procedures can enhance the prognostic outlook for these tumors (5–7). The observed discrepancy between these findings and the prevailing consensus may be attributed to limitations in the study, including a small sample size and an uneven distribution of patients (8–10).

The utilization of data models for analysis and prediction has become increasingly prevalent in the domains of population health assessment and oncology with the advent of big data medicine and precision medicine in today’s world (11, 12). Given the limited occurrence and variances among patients (8), the personalized evaluation of cancer survival duration and tailored therapies for diverse patients assume significant significance in the realm of SCCP. In the realm of rare diseases, the utilization of Surveillance, Epidemiology, and End Results (SEER) databases greatly enhances prognostic evaluations (13). These databases are esteemed for their extensive temporal scope and population-based data. Additionally, nomograms offer physicians a personalized and visually informative approach to prognostic assessment (14). By serving as a visual prediction model, nomograms aid in the comprehensive examination of prognostic risk factors, thereby facilitating treatment decision-making.

To our knowledge, limited research has been conducted on the prognostic assessment of SCCP. Furthermore, there is no comprehensive nomogram for assessing cancer- specific survival (CSS) in patients with SCCP. Therefore, the aim of this research is to evaluate and contrast the predictive features of SCCP in patients identified with small-cell prostate cancer. Prognostic factors will be integrated, using SEER’s multicenter case collection, and a nomogram will be developed to predict the 12- and 24-month CSS of patients. The nomogram’s intuitive and easy-to-use characteristics make it a crucial instrument for promoting precision medicine, as it aids in delivering individualized prognostic forecasts for diverse patients, thereby enhancing the standard of interaction between doctors and SCCP patients.

## Materials and methods

### Study population selection

SEER covers about 30% of the total population in the United States, gathering data on cancer patients from 18 registries. Authors have been granted access to this database (username: 15395-Nov2022).Using the SEER database, individuals diagnosed with SCCP between 2004 and 2015 were identified by referring to the codes provided in the International Classification of Diseases in Oncology, 3rd edition (ICD-O-3).The most recent follow-up occurred in November 2020, with all information being obtained through the utilization of SEER*Stat software (National Cancer Institute, Bethesda, MD, USA, version 8.4.2).We adhered to the recommendations for Transparent Reporting of Multivariate Predictive Models for Individual Prognosis or Diagnosis (15). The SEER database (https://seer.cancer.gov/data/) is a public database with personal identifiers removed, so ethics committee approval and informed patient consent were not required for this study.

### Exclusion and inclusion of data

These histology codes 8041/3 include the primary site code C61.9. Excluded from the study were patients who did not have confirmed diagnoses through histological examination, those with non-primary tumors, individuals diagnosed after death or autopsy, cases with unknown causes of death, and patients with survival times of less than one month after diagnosis. Additionally, tumors without known tumor-node-metastasis (TNM) staging (T0/TX, NX and MX) were not included. The variables considered in the analysis of the data encompassed the following factors: age upon diagnosis, marital status, ethnicity, year of diagnosis, level of severity, TNM stage, Summary stage, PSA levels, surgical intervention, radiation therapy, and chemotherapy. SEER states that the data from 2004 to 2015 align with the 6th edition of TNM.CSS represents the period from the diagnosis to the final follow-up or demise due to primary cancer reasons.

### Model building and validation

We divided 18 registries into 7:3 training and validation cohorts, with 13 Registries in the training cohort and 5 in the validation cohort. The data for the 5 registries in the validation cohort are derived from relatively independent data sets. To identify the factors linked to CSS, we employed Cox proportional-hazards models and Lasso regression. Additionally, we calculated the hazard ratio (HR) and 95% confidence interval (CI) for each factor. To detect independent prognostic factors (P< 0.05), a 10-fold cross-validated Lasso regression was employed to screen prognostic variables identified through multivariate analysis. A 12-month predictive model and a 24-month nomogram were constructed to estimate CSS.

We evaluated the predictive dependability and capability of the nomogram by utilizing the consistency index (C-index), the area beneath the curve (AUC), the receiver operating characteristic curve (ROC), and the calibration curve. Good predictive performance was indicated by a C-index higher than 0.5 and a calibration curve distribution that closely resembled the diagonal. The Bootstrap method was utilized for internal validation, employing 1000 samples. To evaluate the clinical usefulness of the model, a validation cohort was employed along with decision curve analysis (DCA) to assess its applicability.

### Analyses of statistics

Categorical variables were presented as numbers and percentages (%), and continuous variables were expressed as mean (SD). Categorical variable tests were conducted using the χ2 test or Fisher’s exact test. The t-test or u-test was used to compare continuous variables. The Kaplan-Meier method was utilized to conduct survival analysis. R v.4.3.1 statistical software (, The R Foundation, Vienna, Austria) was utilized for all analyses, while comprehensive statistical analysis of the gathered data was carried out using SPSS 25.0 (IBM Corporation, Armonk, NY, USA). Statistical significance was determined when the P-value was less than 0.05 in both directions.

## Results

### Population baseline characteristics

**Figure 1:**
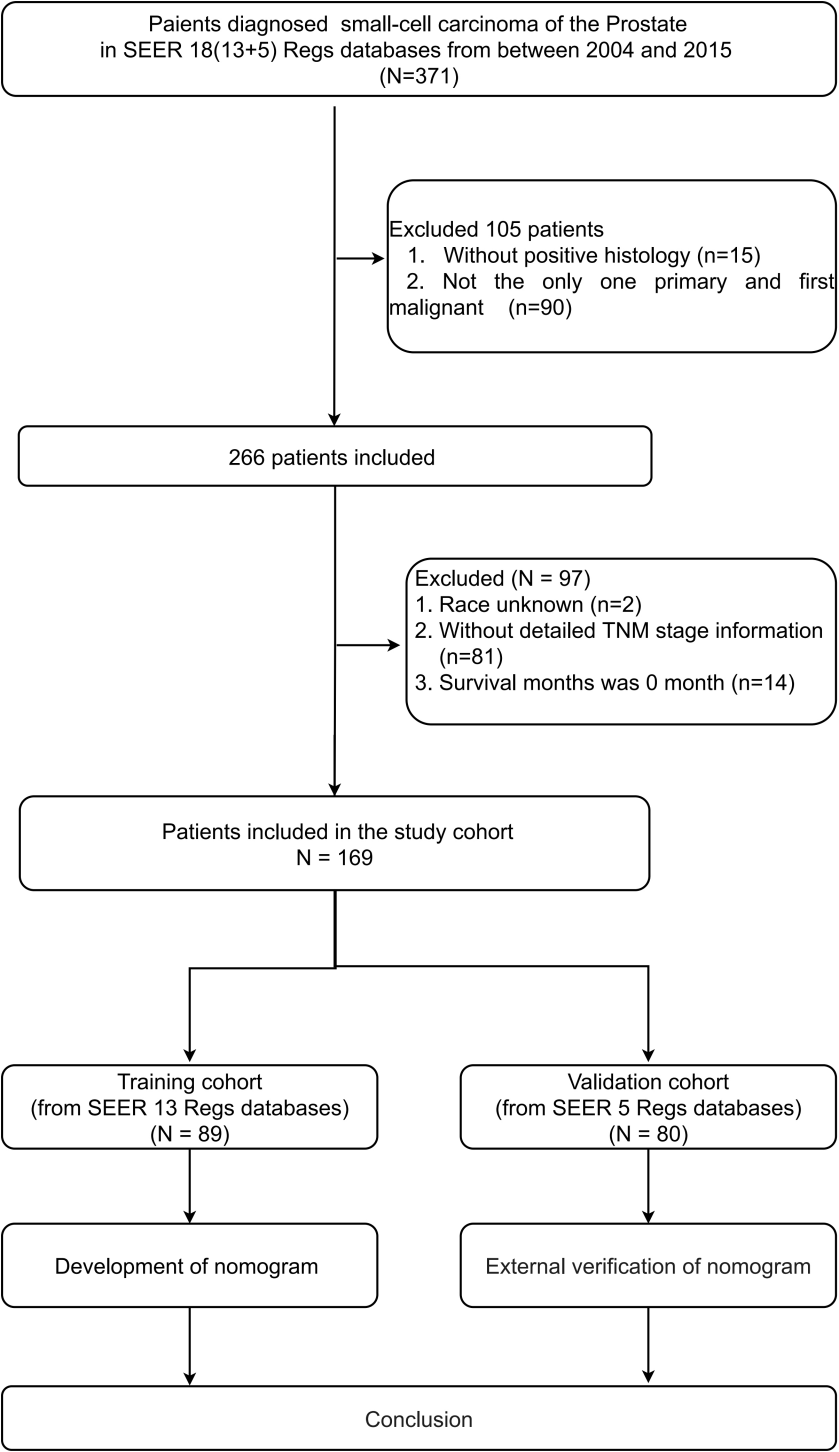
Flow chart for small-cell carcinoma of the prostate patients.

A total of 169 SCCP patients were included in the analysis. For the construction of the nomogram, a total of 89 patients were included in the training cohort, while the validation group consisted of 80 patients. **Figure 1** provides a concise overview of the comprehensive procedure for selecting patients. There were no significant differences in basic information between the training and validation groups, except for race and primary surgery, which showed statistical significance (P < 0.05) for only two variables.

In the training set, the patient attributes were as follows: The average (standard deviation) age was 68.9 ± 11.6 years; 78.7% (70 out of 89) were of Caucasian ethnicity; 43 (48.3%) were diagnosed during the years 2012-2015; 59.6% of the participants were married (53 out of 89); and 64.0% of the patients had undergone chemotherapy. Additionally, the initial diagnosis revealed that a significant proportion of patients (79.8%) were classified as AJCC stage IV, with similar numbers of patients having regional (18.0%) and distant (62.9%) summary stages. In most cases (78 out of 89), the level of prostate-specific antigen (PSA) was either less than 20 ng/ml or not recorded. **Table 1** provides detailed information.

**Table 1:** Baseline characteristics of patients in the training cohort and validation cohort from SEER database.

Characteristic	Total (n = 169)	Training (n = 89)	Validation (n = 80)	*P* value
Age				0.745
<60	38 (22.5)	20 (22.5)	18 (22.5)	
60-74	80 (47.3)	40 (44.9)	40 (50.0)	
≥75	51 (30.2)	29 (32.6)	22 (27.5)	
**Age, Mean± SD**	68.6 ± 11.7	68.9 ± 11.6	68.3 ± 11.8	0.775
**Age, Median (range)**	68.0 (61.0, 77.0)	69.0 (62.0, 78.0)	67.5 (61.0, 75.0)	0.690
Race				0.048
White	144 (85.2)	70 (78.7)	74 (92.5)	
Black	15 (8.9)	11 (12.4)	4 (5.0)	
Other^*^	10 (5.9)	8 (9)	2 (2.5)	
Marital status				0.083
Married	113 (66.9)	53 (59.6)	60 (75)	
Single	23 (13.6)	16 (18)	7 (8.8)	
Other^**^	33 (19.5)	20 (22.5)	13 (16.2)	
Year of diagnosis				0.445
2004-2007	34 (20.1)	17 (19.1)	17 (21.2)	
2008-2011	61 (36.1)	29 (32.6)	32 (40.0)	
2012-2015	74 (43.8)	43 (48.3)	31 (38.8)	
Grade				0.465
II	8 (4.7)	3 (3.4)	5 (6.2)	
III	53 (31.4)	32 (36)	21 (26.2)	
IV	22 (13.0)	10 (11.2)	12 (15.0)	
Unknown	86 (50.9)	44 (49.4)	42 (52.5)	
T stage				0.332
1	26 (15.4)	10 (11.2)	16 (20)	
2	53 (31.4)	32 (36)	21 (26.2)	
3	26 (15.4)	14 (15.7)	12 (15.0)	
4	64 (37.9)	33 (37.1)	31 (38.8)	
N stage				0.218
0	95 (56.2)	54 (60.7)	41 (51.2)	
1	74 (43.8)	35 (39.3)	39 (48.8)	
M stage				0.569
0	68 (40.2)	34 (38.2)	34 (42.5)	
1	101 (59.8)	55 (61.8)	46 (57.5)	
AJCC stage				0.714
II	30 (17.8)	17 (19.1)	13 (16.2)	
III	3 (1.8)	1 (1.1)	2 (2.5)	
IV	136 (80.5)	71 (79.8)	65 (81.2)	
Summary stage				0.525
Local	30 (17.8)	17 (19.1)	13 (16.2)	
Regional	36 (21.3)	16 (18)	20 (25.0)	
Distant	103 (60.9)	56 (62.9)	47 (58.8)	
PSA				0.752
<10	52 (30.8)	25 (28.1)	27 (33.8)	
10-20	9 (5.3)	6 (6.7)	3 (3.8)	
≥20	19 (11.2)	11 (12.4)	8 (10.0)	
Unknown	89 (52.7)	47 (52.8)	42 (52.5)	
Chemotherapy				0.123
No/Unknown	52 (30.8)	32 (36.0)	20 (25)	
Yes	117 (69.2)	57 (64.0)	60 (75)	
Surgery				0.032
No	121 (71.6)	70 (78.7)	51 (63.7)	
Yes	48 (28.4)	19 (21.3)	29 (36.2)	
Radiation				0.233
No/Unknown	104 (61.5)	51 (57.3)	53 (66.2)	
Yes	65 (38.5)	38 (42.7)	27 (33.8)	

^*^American Indian/AK Native and Asian/Pacific Islander; ^**^divorced, separated, and widowed.

### Prognostic factors analysis

A 12-month CSS of 44.6% and a 24-month CSS of 25.5% are illustrated in **Supplementary Figure 1**.

**Figure 2:**
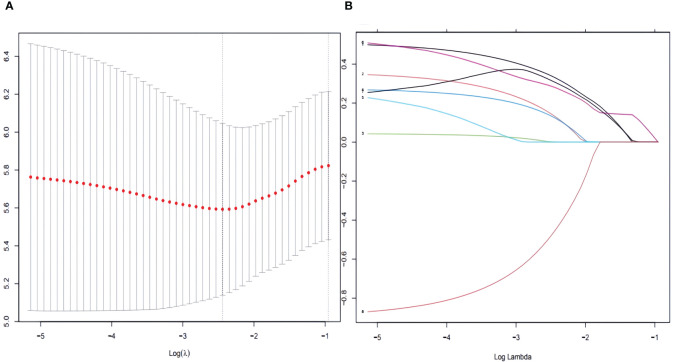
To further narrow the range of variables involved in the regression analysis, the parameters were adjusted by 10-fold cross-validation, and established using the least absolute shrinkage and selection operator (LASSO) in the Cox model in the training set **(A)**. Combining the distribution of LASSO coefficients for eight variables (age, race, grade, N stage, M stage, AJCC stage, Summary stage and chemotherapy) in SCCP patients, an optimal lambda filter was used to generate four variables (age, race, Summary stage and chemotherapy) with non-zero coefficients **(B)**.

**Table 2** shows that patients with AJCC stage (IV), Summary stage (distant), and those who did not receive chemotherapy performed unfavorably in both univariate and multivariate Cox regression analysis (HR>1, P< 0.05).To enhance the screening process, we incorporated differentiation grades into Lasso regression (**Figure 2**). According to **Table 2**, four variables were identified as independent prognostic factors (P<0.05) through multivariate proportional-hazards regression and Lasso regression analyses.

**Table 2:** Multivariate analyses for CSS in patients with SCCP.

Characteristic	Univariate	*P* value	Multivariate	*P* value
	HR (95% CI)		HR (95% CI)	
Age				
<60	Ref		Ref	
60-74	1.24 (0.68~2.27)	0.491	1.19 (0.51~2.79)	0.694
≥75	2.36 (1.27~4.39)	0.007	2.05 (0.92~4.56)	0.077
Race				
White	Ref		Ref	
Black	0.66 (0.30~1.44)	0.293	0.53 (0.19~1.48)	0.228
Other^*^	3.02 (1.42~6.44)	0.004	3.97 (1.50~10.5)	0.006
Marital status				
Married	Ref		Ref	
Single	0.84 (0.44~1.61)	0.595	0.73 (0.31~1.69)	0.459
Other^**^	1.33 (0.73~2.44)	0.356	0.95 (0.38~2.39)	0.919
Year of diagnosis				
2004-2007	Ref		Ref	
2008-2011	0.84 (0.44~1.61)	0.595	0.72 (0.31~1.67)	0.449
2012-2015	1.33 (0.73~2.44)	0.356	0.95 (0.38~2.37)	0.907
Grade				
II	Ref		Ref	
III	5.18 (0.70~38.18)	0.107	1 (0.11~9.37)	0.997
IV	6.92 (0.88~54.41)	0.066	1.75 (0.15~19.7)	0.652
Unknown	6.48 (0.88~47.56)	0.066	1.46 (0.15~14.55)	0.746
T stage				
1	Ref		Ref	
2	1.23 (0.53~2.84)	0.630	1.12 (0.44~2.9)	0.808
3	1.45 (0.57~3.7)	0.435	0.83 (0.27~2.54)	0.750
4	2.09 (0.91~4.79)	0.083	1.3 (0.48~3.53)	0.601
N stage				
0	Ref		Ref	
1	1.83 (1.14~2.93)	0.012	1.27 (0.71~2.27)	0.413
M stage				
0	Ref		Ref	
1	2.21 (1.35~3.63)	0.002	0.21 (0.02~2.57)	0.221
AJCC stage				
II	Ref		Ref	
III	0 (0~Inf)	0.995	0 (0~Inf)	0.997
IV	2.97 (1.41~6.25)	0.004	4.12 (1.43~11.88)	0.009
Summary stage				
Local	Ref		Ref	
Regional	2.19 (0.88~5.45)	0.092	3.69 (1.26~10.82)	0.017
Distant	3.11 (1.46~6.63)	0.003	44.12 (3.60~541.26)	0.003
PSA				
<10	Ref		Ref	
10-20	0.82 (0.31~2.18)	0.692	1.32 (0.35~4.94)	0.679
≥20	0.66 (0.3~1.44)	0.299	0.54 (0.21~1.43)	0.217
Unknown	0.69 (0.41~1.16)	0.162	0.49 (0.22~1.08)	0.079
Chemotherapy				
No/Unknown	Ref		Ref	
Yes	0.82 (0.5~1.34)	0.42	0.23 (0.11~0.48)	<0.001
Surgery				
No	Ref		Ref	
Yes	0.73 (0.4~1.33)	0.302	0.81 (0.4~1.66)	0.574
Radiation				
No/Unknown	Ref		Ref	
Yes	0.88 (0.56~1.38)	0.574	1.31 (0.72~2.4)	0.384

^*^American Indian/AK Native and Asian/Pacific Islander; ^**^divorced, separated, and widowed.

CSS, cancer-specific survival; SCCP, small-cell carcinoma of the prostate.

### Nomogram construction and validation

**Figure 3:**
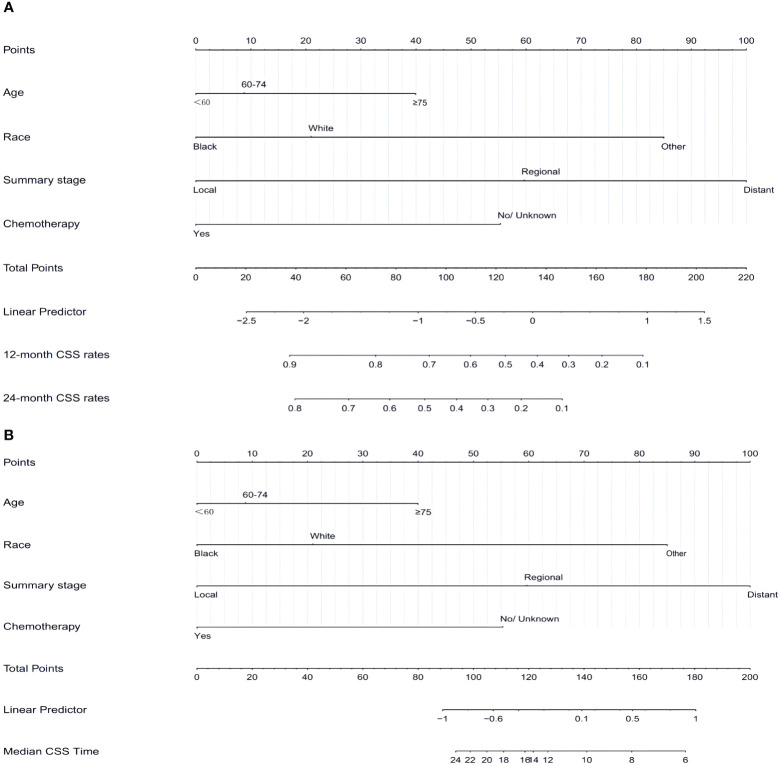
Nomogram **(A, B)** predicting 12-month,24-month, and median CSS of patients with SCCP based on 4 prognostic factors. CSS, cancer-specific survival; SCCP, small-cell carcinoma of the prostate.

Based on the independent prognostic factors identified in the aforementioned multifactorial regression, a nomogram was constructed. By combining the characteristics of each patient, the corresponding scores for each variable were determined. In conclusion, the CSS for 12 months and 24 months was calculated by adding up the variables, evaluating the chances of survival and median CSS for various individuals, as outlined in **Figure 3**.

**Figure 4:**
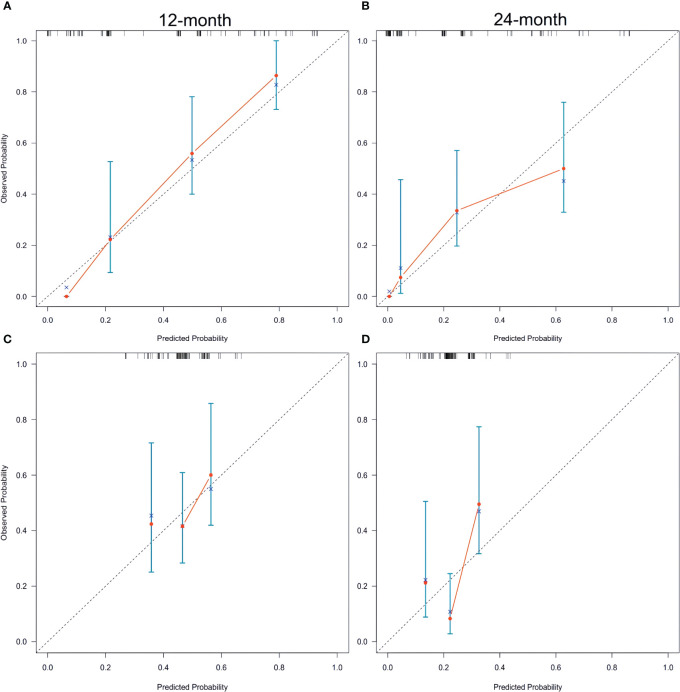
Calibration plots of the nomogram for 12-, and 24-month CSS prediction of the training set **(A, B)** and verification set **(C, D)**. X-axis represents the nomogram-predicted probability of survival; Y-axis represents the actual CSS probability. A perfectly accurate nomogram prediction model would result in a plot that the observed and predicted probabilities for given groups fall along the 45-degree line. Dots with bars represent nomogram-predicted probabilities along with 95% confidence interval. CSS, cancer-specific survival; SCCP, small-cell carcinoma of the prostate.

**Figure 5:**
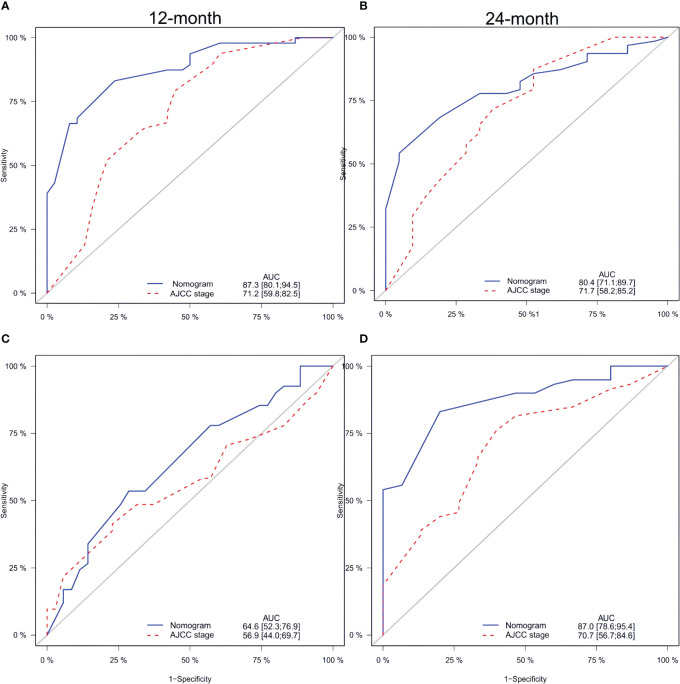
Comparison of the ROC curves of the nomogram and the TNM stage system for 12-, and 24- month CSS prediction in the training set **(A, B)**. And the ROC curves of the nomogram for 12-, and 24-month CSS prediction in the verification set **(C, D)**. CSS, cancer-specific survival.

In both the training and validation sets, the calibration plot for CSS probability at 12 months and 24 months showed agreement (**Figure 4**). The C-index values for the training set were 0.768 (95% CI, 0.721-0.815), while for the validation set they were 0.661 (95% CI, 0.581-0.740), indicating a strong agreement between the actual and nomogram-predicted CSS. The ROC curves for 12 months and 24 months were compared between the nomogram and AJCC stage, revealing AUC values of 87.3% versus 71.2% and 80.4% versus 71.7%, respectively (**Figures 5A, B**).

**Figure 6:**
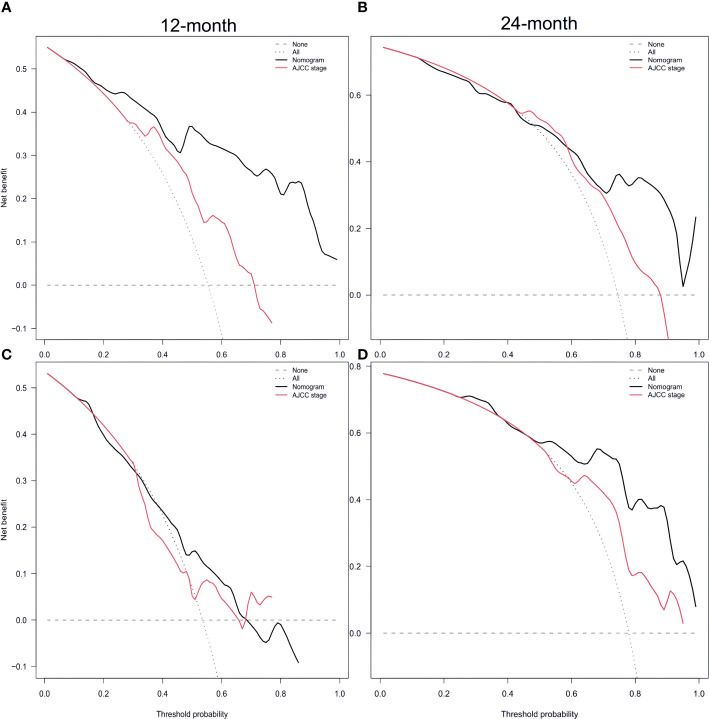
Decision curve analysis of training set **(A, B)** and validation set **(C, D)** compare with the AJCC stage for predicting 12 months CSS and 24 months CSS. CSS, cancer-specific survival **Supplementary Figure 1**.

Furthermore, **Figures 5C** and **D** display the calibration plot for external validation. The AUC for 12-month and 24-month was (64.6% vs. 56.9%) and (87.0% vs. 70.7%) respectively. Based on a decision curve analysis (DCA), the nomogram demonstrates superior accuracy in predicting CSS compared to the AJCC staging system (**Figure 6**).

### Nomograms in clinical practice

A nomogram can be created by calculating the sum of individual variable scores, which can then be used to predict the survival rates at 12 and 24 months for every patient. Take into account a Caucasian patient who is 65 years old and has regional stage, but is apprehensive about undergoing chemotherapy. In conjunction with the myth, the overall score amounted to approximately 145 compared to 90 (chemotherapy versus non-chemotherapy). If this patient had received chemotherapy, the CSS would have been approximately 70%-75% and 50%-52% at 12 months and 24 months, respectively. The median CSS time with or without chemotherapy for this patient was about 24 months vs. 9 months. The CSS should be prolonged with chemotherapy in patients with SCCP. For patients of other race, even if diagnosed early and young, prognosis is poor without systematic treatment, with the median CSS of only 9 months. This highlights the importance of addressing medical treatment for ethnic minorities.

## Discussion

Pure prostate small cell carcinoma (PSCC) is an uncommon prostate tumor that exhibits an exceptionally high level of malignancy. Despite its rarity, there has been a noticeable upward trend in its incidence over the years. From 2012 to 2015, the number of newly diagnosed patients more than doubled compared to the period from 2004 to 2007, consistent with previous studies. This increase can be attributed to advances in diagnostic techniques, such as improved immunohistochemistry and genomics, enhancing SCCP diagnosis accuracy (16, 17). Notably, INSM1 has emerged as a sensitive and specific marker for SCCP identification (18).The biomarkers P501S and PSMA demonstrate greater reliability in discerning SCCP source compared to PSA (19).

The uncertainty surrounding the mechanism by which age functions as an independent prognostic factor requires further investigation (20). It is crucial to explore whether the unfavorable prognosis associated with older patients can be attributed to the heightened aggressiveness of tumor biology or is merely a consequence of their diminished physical condition and suboptimal chemotherapy. Racial disparities in prognoses emphasize the need to implement evidence-based strategies and ensure equitable access to resources, particularly in communities of color, to mitigate these disparities (21). The AJCC system, while founded on extensive data regarding prostate cancer, appears to lack efficacy in accurately staging SCCP. Furthermore, the overall staging performance in SCCP is comparable to that seen in small cell lung cancer.

Similar to previous research (22), PSA screening for prostate cancer may have some benefits, but its efficacy was limited in the context of SCCP (23). In cases of pure small cell carcinoma of the prostate (SCCP), where there is no disruption of follicular structure, serum PSA levels remain unaltered even in the presence of extensive metastases. Conversely, patients with a combination of small cell carcinoma and adenocarcinoma may exhibit elevated serum PSA levels, indicating limited diagnostic utility and lack of specificity for SCCP. Our study further revealed that among patients with available PSA data, 65% had PSA levels below 10 ng/ml, 76.25% had levels below 20 ng/ml, and only 23.7% had levels exceeding 20 ng/ml.

Although chemotherapy is a standard treatment option (24, 25), it has yielded unsatisfactory results (26). The development of new chemotherapeutic agents lacks momentum (27), and chemotherapy regimens using doxorubicin, etoposide, and cisplatin have some activity but high treatment-related toxicity (3). Their main shortcomings include a small sample size, low recruitment rate, and high treatment-related adverse events. The primary reason this study did not find radiotherapy and surgery to improve survival is that most patients were in advanced stages, consistent with previous studies. The significance of localized treatment was demonstrated in the National Cancer Database (NCDB) (5, 28, 29). It is evident that patients can derive greater benefits through early detection alone (16, 30). The efficacy of localized therapy depends heavily on the effectiveness of systemic chemotherapy (31).

The development of a prognostic assessment tool provides the benefit of offering an initial intuitive estimation of survival, enabling collaborative decision-making on appropriate treatment options for clinicians and patients s (32).However, it is important to emphasize that prognostic tools should not replace clinical judgment. Clinicians must consider individual variations, such as the severity of comorbidities and physical condition, when making decisions. While numerous predictive models exist for prostate cancer (33, 34) and neuroendocrine prostate cancer (35), our nomogram is specifically designed for pure SCCP with well-defined staging, eliminating potential data bias resulting from unclear staging. To fill this void, we created a training dataset that achieved a C-index of 0.768 ± 0.024, and a validation dataset with a C-index of 0.661 ± 0.041, respectively. These datasets were built by incorporating information from the SEER database (36–38) and building upon the findings of the prior study. New insights for clinical application can be obtained by constructing nomograms that predict overall survival at 1 year and 2 years.

In this study, it is necessary to take into account various constraints, including its retrospective nature. Our dataset did not allow the examination of chemotherapy, patient performance status, or co-morbidities, all of which could impact survival outcomes in cancer patients. In contrast to studies conducted at a single center, the precision of staging and pathologic diagnoses in national registries can differ greatly among different institutions. However, we addressed this limitation by focusing on measuring prostate cancer-specific survival rather than overall survival. This study has notable strengths, including the use of a population-based design and a substantial sample size, both of which are highly relevant in analyzing infrequent neoplasms.

## Conclusions

CSS in SCCP was influenced by factors such as age, ethnicity, Summary stage, and the administration of chemotherapy. The nomogram for predicting CSS in SCCP was established in our pioneering population-based study on these risk factors. The nomogram exhibited excellent precision and practicality in both internal and external validation, showcasing its accuracy and clinical usefulness. It demonstrates superior potential utilization compared to traditional TNM staging and can assist healthcare professionals in making well-informed choices, offering a personalized prognostic benchmark for individuals with SCCP.

## Data availability statement

The original contributions presented in the study are included in the article/**Supplementary Material**. Further inquiries can be directed to the corresponding authors.

## Author contributions

YD: Writing – original draft, Writing – review & editing. JS: Conceptualization, Investigation, Software, Writing – review & editing. ZS: Data curation, Writing – review & editing. YW: Methodology, Supervision, Writing – original draft. LM: Project administration, Resources, Validation, Visualization, Writing – review & editing.

## Supplementary material

Supplementary Table 1 Model variable screening using lasso regression.

Supplementary Table 2 Model variable screening using stepwise Selection.

Supplementary Table 3 Model variable screening using best subset.
