# Supplementary figures and images for: Prognostic nomogram to predict cancer-specific survival with small-cell carcinoma of the prostate: a multi-institutional study
Source: Front Oncol. 2024 May 10;14:1349888. doi: 10.3389/fonc.2024.1349888 (PMC11116562; doi:10.3389/fonc.2024.1349888)

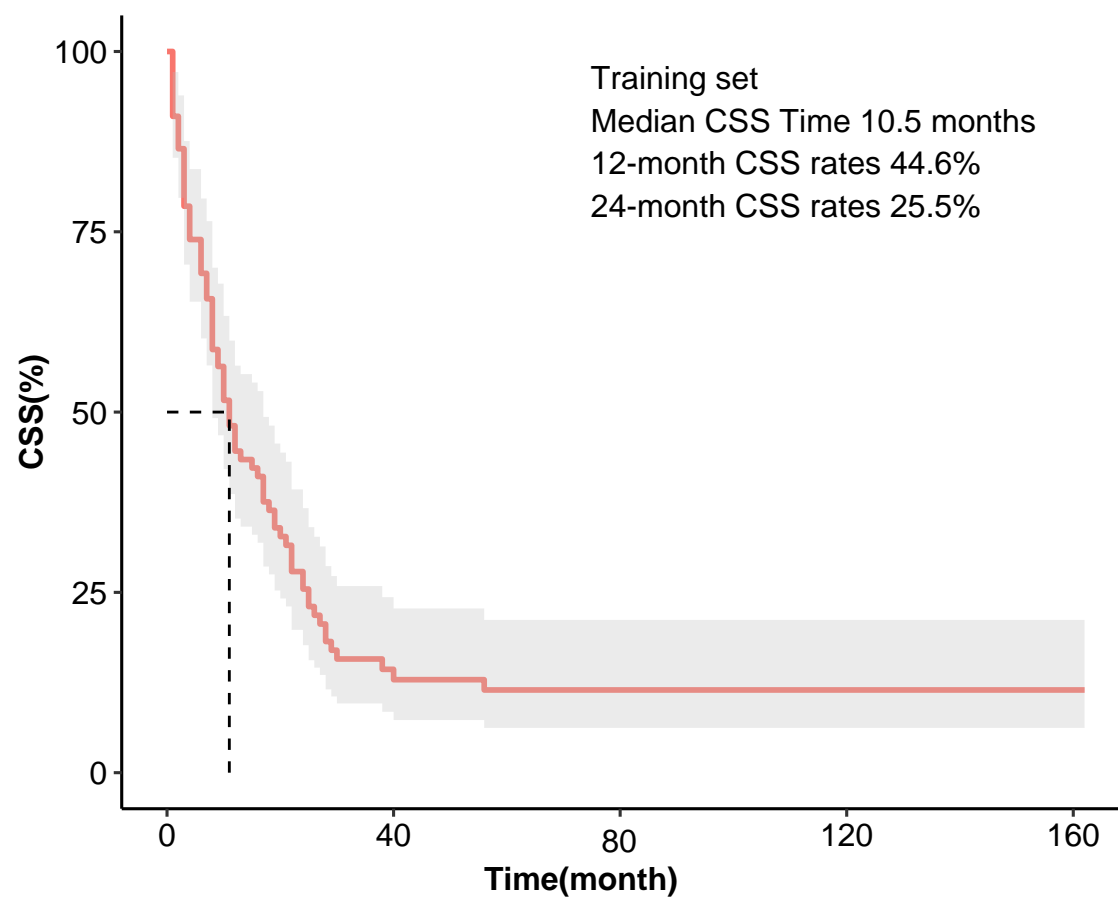

Supplement: Supplementary file 1 [file Image_1.pdf]
