# Supplementary material for: Prognostic nomogram to predict cancer-specific survival with small-cell carcinoma of the prostate: a multi-institutional study
Source: Front Oncol. 2024 May 10;14:1349888. doi: 10.3389/fonc.2024.1349888 (PMC11116562; doi:10.3389/fonc.2024.1349888)
Supplement: Supplementary Table 1 — Model variable screening using lasso regression. [file Table_1.docx]

**Supplementary Table 1. Model variable screening using lasso regression**

| **Variable.name** | **Coefficient** | **lambda.type** |
| --- | --- | --- |
| **Age** | 0.309 | lambda.min |
| **Race** | 0.2 | lambda.min |
| **Radiation** |  | lambda.min |
| **Chemotherapy** | -0.469 | lambda.min |
| **Marital** |  | lambda.min |
| **Year.of.diagnosis** |  | lambda.min |
| **Grade** | 0.028 | lambda.min |
| **T stage** | 0.003 | lambda.min |
| **N stage** | 0.197 | lambda.min |
| **M stage** |  | lambda.min |
| **Summary stage** | 0.537 | lambda.min |
| **Surgery** | -0.197 | lambda.min |

**Supplementary Table 2. Model variable screening using stepwise Selection**

| **Variable** | **Estimate** | **HR** | **Std Error** | **z value** | **P value** |
| --- | --- | --- | --- | --- | --- |
| **Age** |  |  |  |  |  |
| ＜60 | Ref |  |  |  |  |
| 60-74 | 0.123 | 1.131 | 0.327 | 0.377 | 0.706 |
| ≥75 | 0.664 | 1.942 | 0.311 | 2.132 | 0.033 |
| **Race** |  |  |  |  |  |
| White | Ref |  |  |  |  |
| Black | -0.416 | 0.66 | 0.435 | -0.958 | 0.338 |
| Other | 1.47 | 4.351 | 0.447 | 3.288 | 0.001 |
| **Chemotherapy** |  |  |  |  |  |
| No/Unknown | Ref |  |  |  |  |
| Yes | -1.071 | 0.343 | 0.334 | -3.211 | 0.001 |
| **Summary stage** |  |  |  |  |  |
| Local | Ref |  |  |  |  |
| Regional | 1.076 | 2.934 | 0.477 | 2.255 | 0.024 |
| Distant | 2.142 | 8.514 | 0.424 | 5.048 | 0 |
| **Radiation** |  |  |  |  |  |
| No/Unknown | Ref |  |  |  |  |
| Yes | 0.435 | 1.544 | 0.26 | 1.671 | 0.095 |

**Supplementary Table 3. Model variable screening using best subset**

| **Item** | **Number of Variables** |
| --- | --- |
| max.R.squared | 8 |
| max.Adjusted.R.squared | 8 |
| min.BIC | 4 |
| min.Mallows.Cp | 6 |
